# Using light to image millimeter wave based on stacked meta-MEMS chip

**DOI:** 10.1038/s41377-024-01733-6

**Published:** 2025-01-22

**Authors:** Han Wang, Zhigang Wang, Cheng Gong, Xinyu Li, Tiansheng Cui, Huiqi Jiang, Minghui Deng, Bo Yan, Weiwei Liu

**Affiliations:** 1 Institute of Modern Optics, Nankai University, Tianjin Key Laboratory of Micro-scale Optical Information Science and Technology, Tianjin, 300350 China; 2 School of Electronic Science and Engineering, University of Electronic Science and Technology of China, Chengdu, 611731 China

**Keywords:** Metamaterials, Imaging and sensing

## Abstract

A stacked metamaterial MEMS (meta-MEMS) chip is proposed, which can perfectly absorb electromagnetic waves, convert them into mechanical energy, drive movement of the optical micro-reflectors array, and detect millimeter waves. It is equivalent to using visible light to image a millimeter wave. The meta-MEMS adopts the design of upper and lower chip separation and then stacking to achieve the “dielectric-resonant-air-ground” structure, reduce the thickness of the metamaterial and MEMS structures, and improve the performance of millimeter wave imaging. For verification, we designed and prepared a 94 GHz meta-MEMS focal plane array chip, in which the sum of the thickness of the metamaterial and MEMS structures is only 1/2500 wavelength, the pixel size is less than 1/3 wavelength, but the absorption rate is as high as 99.8%. Moreover, a light readout module was constructed to test the millimeter wave imaging performance. The results show that the response speed can reach 144 Hz and the lens-less imaging resolution is 1.5 mm.

## Introduction

Millimeter waves have excellent penetration characteristics^1–3^, allowing for easy “perspective” of smoke, fog, plastic sheets, clothing, and other obstacles that cannot be penetrated by visible or infrared light^4,5^. This feature can be used in important fields such as security checks^6–9^, biomedicine^10–13^, military and national defense^14–17^. In the millimeter wave band, 94 GHz has a shorter wavelength that can achieve higher imaging resolution. Meanwhile, 94 GHz is located in the atmospheric transmission window, and the loss during transmission and imaging is small, which is conducive to improving the imaging signal-to-noise ratio. The array detectors are the key to achieving millimeter wave imaging. They can achieve high-speed imaging without scanning, thus becoming a research hotspot. In general, the millimeter wave array detectors mainly include: the receiver array^18–20^, the pyroelectric array^21^, the direct detection antenna array^22^, and so on. In general, these detectors are based on the electromagnetic wave-electrical signal conversion mechanism. Their pixels need wires to connect, and the signals need wires to lead out. The larger the array, the more complex the wiring, and the more difficult the detector design.

In recent years, metamaterial technology has been rapidly developed. As an artificial microstructure periodic array composite material, metamaterials can provide unprecedented ability to manipulate electromagnetic waves by changing the geometric shape and size of the structural units. Many outstanding achievements or applications based on metamaterials have been proposed, including negative refraction^23,24^, active terahertz regulation^25^, cloak^26^, perfect absorber^27^, metalens^28^, and so on. Micro-electro-mechanical system (MEMS) belongs to a typical interdisciplinary research field^29–31^. MEMS-based devices have the advantages of small size, lightweight, low energy consumption, small inertia, high resonant frequency, and short response time^32–39^. They have broad application prospects in fields such as electronics, communication, and sensing.

In the field of electromagnetic wave detection and imaging, a novel array detection mechanism combining metamaterials and MEMS has been proposed. It converts the electromagnetic waves into micromechanical deflection through metamaterial absorbing and execution structure, and then reads them out through visible light devices, which has the advantages of non-contact, parallel, high speed, and so on. The detectors based on this mechanism have been applied in fields such as terahertz and infrared detection. In 2011, H. Tao et al. designed, fabricated, and characterized metamaterial-enhanced MEMS cantilever pixels for far-infrared detection^40^. In 2013, F. Alves et al. proposed a THz bi-material MEMS sensor with a metamaterial absorber^41^. In 2016, H. Bilgina et al. reported a MEMS-based terahertz detector with a metamaterial-based absorber and optical interferometric readout^42^. In 2017, Y. Wen et al. proposed a photomechanical MEMS meta-molecule array for real-time THz imaging at a frame rate of 20 Hz^43^. In 2018, F. Alves et al. developed a MEMS terahertz-to-infrared converter using frequency selective planar metamaterial for THz imaging^44^. In May 2022, H. Zhu et al. reported a metasurface-based terahertz optomechanical MEMS detector, which obtains absorptivity higher than 90% from 3.24 to 3.98 THz^45^. In August 2022, Z. Li et al., demonstrated the design and fabrication of substrate-free Au/SiNx/Au MEMS Metafilm for THz sensing^46^. In November 2022, H. Ozpinar et al. proposed a penta‑band metamaterial MEMS absorber for THz sensing and imaging^47^.

In this paper, we focus on millimeter wave imaging and propose a stacked meta-MEMS chip. It is based on the design of stacked upper and lower chips, achieving a structure of “dielectric-resonant-air-ground”. The upper chip consists of the dielectric layer and metamaterial resonant layer, which forms the MEMS cantilever structure and micro-reflector array. The lower chip includes an air layer and a ground layer. This paper will explore the design, fabrication, and testing methods of the meta-MEMS to achieve more sensitive and faster millimeter-wave imaging. Meanwhile, the design idea and imaging method adopted will be able to serve as a promising advanced technology platform for a wide range of millimeter wave detection and imaging applications.

## Results

### Design and simulation

**Fig. 1:**
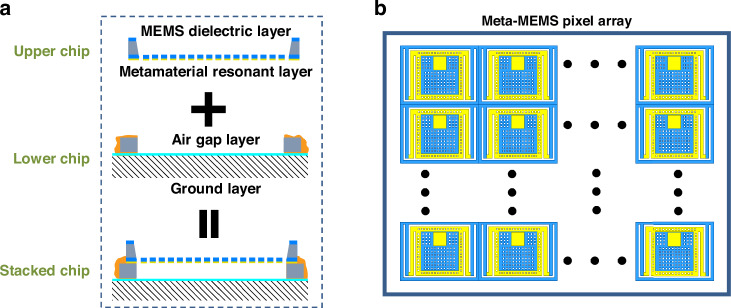
Stacked chip’s schematic. **a** Schematic diagram of the stacked meta-MEMS chip; **b** Diagram of the meta-MEMS pixel array

Figure 1a presents the schematic diagram of the meta-MEMS, which is composed of stacked upper and lower chips. The upper chip includes a metamaterial resonant layer and a MEMS dielectric layer, while the lower chip consists of an air gap layer and a ground layer. The metamaterial resonant layer can efficiently absorb millimeter waves with the cooperation of other layers, and convert it into the micromechanical energy of the bi-material cantilevers in the MEMS structure to drive the micro-reflector array to achieve the corresponding deflection. The thickness of the air gap layer can help regulate the frequency of the absorption peak, and the ground layer ensures the transmittance of millimeter waves is close to zero, thus improving the absorption. The meta-MEMS adopts the design of upper and lower chip separation and then stacking to achieve the “dielectric-resonant-air-ground” structure, which reduces the thickness of the metamaterial and MEMS layer, thus improving the performance (sensitivity and response time). Figure 1b shows a schematic diagram of the meta-MEMS pixel array for detection and imaging.

**Fig. 2:**
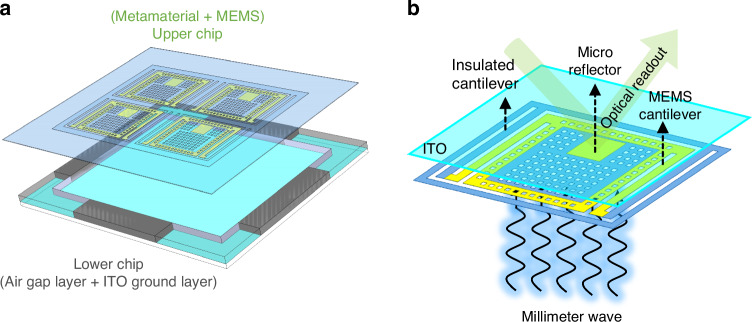
Stacked pixels’ schematic. **a** 2 × 2 pixels model of the lower and upper chips before stacking; **b** Schematic diagram of a meta-MEMS pixel

Figure 2a depicts a 2 × 2 pixel model of the lower and upper chips before stacking, and Fig. 2b shows the schematic diagram of a meta-MEMS pixel. The lower chip is composed of ITO-coated quartz glass and silicon support frames. The silicon frame consists of four silicon customized thickness support blocks. The ITO square resistance is 6 Ω, which can be used as the ground layer to block the transmission of millimeter waves. Meanwhile, an air gap layer could be formed through the silicon support frame. The upper chip is composed of metamaterial resonators, MEMS structures (micro-reflectors and cantilevers), and weight-reduction holes. It consists of two layers, namely the silicon nitride (SiNx) dielectric layer and the Au pattern layer. The SiNx plays both a supporting and thermal isolation role in MEMS structure. The Au pattern layer serves as an electric resonance layer, which can regulate the equivalent dielectric constant *ε*. After stacking, the upper and lower chips form a magnetic resonance cavity, which can regulate the equivalent permeability *µ*. In addition, due to the significant difference in thermal expansion coefficients between SiNx and Au, the bi-material cantilevers composed of these two materials form MEMS micro-actuators. They can drive the micro-reflectors to deflect, which is the foundation for achieving visible light readout. Due to the combined effect of electric resonance and magnetic resonance of the stacked chip, the equivalent permeability and dielectric constant can be simultaneously regulated, achieving impedance matching *z* = (*µ*/*ε*)^1/2^, thus perfectly absorbing incident millimeter waves. Then convert them into mechanical energy of the bi-material cantilevers, drive the movement of visible light micro-reflector arrays, and detect millimeter waves through light readout.

The meta-MEMS could be regarded as an equivalent composite material. Due to the complex sub-wavelength structure, it is difficult to accurately calculate the absorption spectrum using analytical methods. Therefore, a numerical calculation method is used. Firstly, a three-dimensional physical model of meta-MEMS pixel is established in an electromagnetic simulation software, and the port, frequency, boundary conditions, and simulation algorithm are set. Then the S-parameters are simulated to obtain the reflection coefficient (*S*_11_) and transmission coefficient (*S*_21_). Finally, the absorption spectrum can be calculated by:

1 $$A=1-R-T=1-{|{S}_{11}|}^{2}-{|{S}_{21}|}^{2}$$

Here, |*S*_11_|^2^ and |*S*_21_|^2^ represent the reflectance *R* and transmission *T*, respectively. To maximize the absorption *A*, the reflection and transmission should be minimized. Because of the blocking of the chip’s ground layer, its transmission is 0. Therefore, the absorptivity can be obtained by *A* = 1- *R*.

**Fig. 3:**
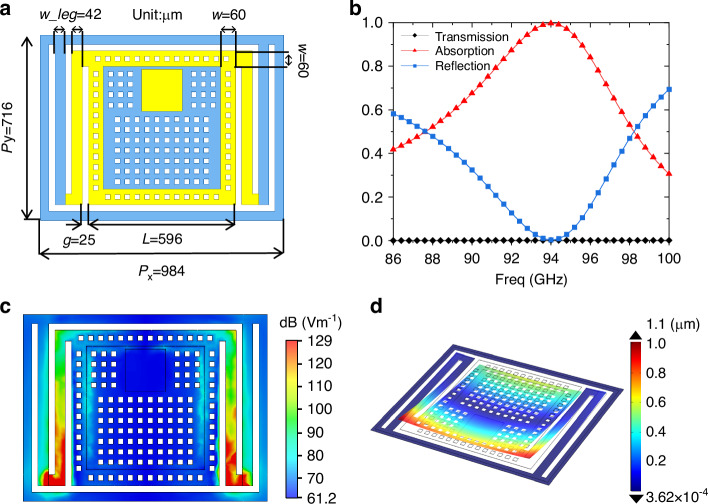
Model and simulation. **a** The model and optimal size of one pixel of the meta-MEMS chip; **b** The simulated spectra; **c** Electric field distribution of the resonant structure; **d** Schematic diagram of the pixel deflection

Next, we designed and optimized a 94 GHz stacked meta-MEMS using electromagnetic simulation software CST. Figure 3a depicts the simulation model and the optimal size of one pixel. The thickness of the meta-MEMS structure is 1.2 µm. In the simulation, the tetrahedral meshing and frequency domain solver based on the finite element method were used, and the absorption spectra obtained were shown in Fig. 3b. When optimizing the structure size, we adopted the idea of first rough simulation and then fine simulation. The specific optimization method is described in detail in the supplementary document S1. It can be seen that the absorptivity reaches 99.9% at 94 GHz, which is close to perfect absorption. Figure 3c is the simulated electric field distribution of one stacked meta-MEMS pixel. It can be shown that the resonant structure of meta-MEMS produces a strong electrical resonance at the bi-material cantilevers. Therefore, the resonant electric field energy is mainly concentrated on the bi-material cantilevers, which helps to efficiently convert electromagnetic energy into mechanical energy. We used the MPHYSICS module in CST to simulate the deformation of a single pixel, as shown in Fig. 3d. Theoretically, it can be obtained by simulation that the end deflection of the bi-material cantilever is about 1.1 µm at 1 K temperature rise.

**Fig. 4:**
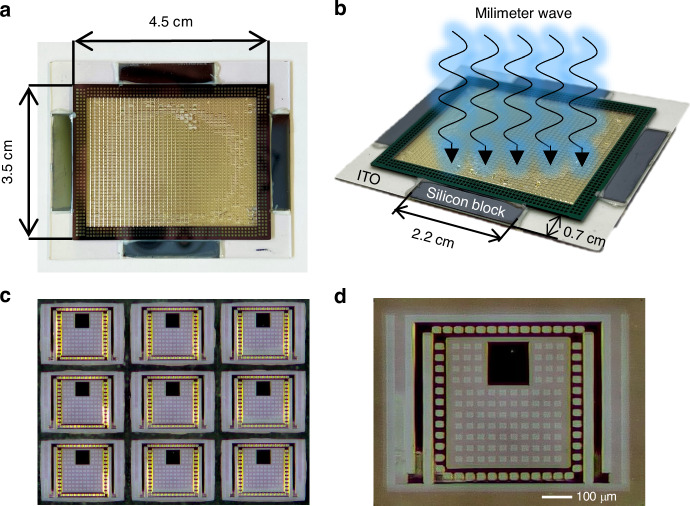
Photos of the chip. **a** The fabricated stacked meta-MEMS array chip; **b** A photo of the chip; **c** A micrograph of 3 × 3 pixels array area; **d** A micrograph of one pixel

Figure 4a shows a photograph of the fabricated meta-MEMS array chip. Its overall size is 3.5 × 4.5 cm; the pixel number of the chip is 40 × 40 = 1600. Due to the use of ITO glass substrate, it is transparent, and visible light readout imaging can be performed from the back of the substrate. Figure 4b depicts a photo of the chip and the size of the silicon support blocks. Figure 4c is a micrograph of 3 × 3 pixels array area in the chip. Figure 4d shows a micrograph of one pixel. The x-y size of each pixel is 984 × 716 µm, which is less than 1/3 of the operating wavelength, theoretically enabling higher resolution. Moreover, due to the stacked design, the thickness of the meta-MEMS structure is only 1.2 microns, which is about 1/2500 of the operating wavelength. It should be mentioned that if the coating and etching process is further improved, the thickness could be further reduced.

### Absorption performance of the meta-MEMS chip

**Fig. 5:**
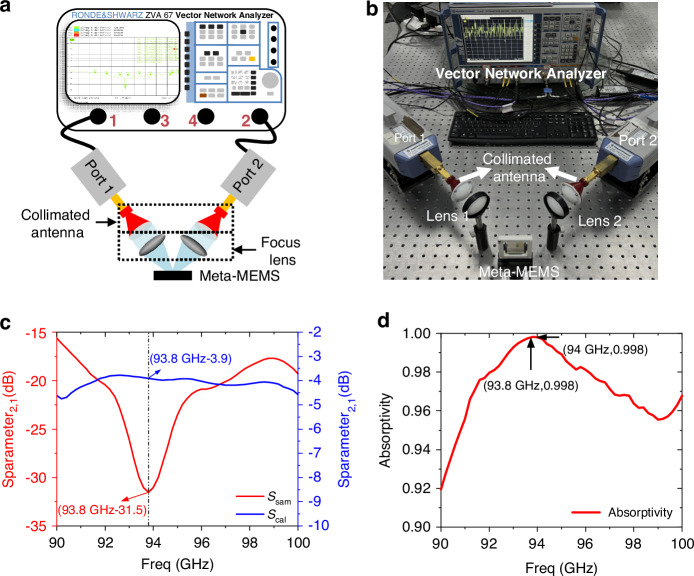
Spectral measurement. **a** Schematic of the reflective spectroscopy measurement system; **b** Photograph of the system; **c** The corresponding S-parameter curves; **d** The measured absorption spectrum of the chip

Then, a reflective spectroscopy measurement system based on a vector network analyzer is built to test the absorption spectrum of the meta-MEMS chip. Figure 5a shows the schematic diagram of the system and Fig. 5b is the corresponding photo of the system. The specific optical path is as follows: the output antenna (Port 1) of the vector network analyzer emits millimeter wave radiation and is then focused onto the sample by a focusing lens. Then, the radiation reflected from the meta-MEMS is focused onto the receiving antenna (Port 2) of the vector network analyzer through a second lens to obtain information about the reflected millimeter wave.

The detailed steps for measuring the absorption spectrum can be described as follows: The first is to measure the S-parameter *S*_21_ of a reference object with near 100% reflectivity (Au mirror) as calibration data *S*_cal_. The second step is to measure the S parameter S_21_ of the meta-MEMS chip as sample data *S*_sam_. Figure 5c shows the corresponding S-parameter curves measured by the system. The third step is to subtract the two S-parameters from each other, and the target’s S parameter is *S* = *S*_sam_ − *S*_cal_. The fourth step, the absorption spectrum can be obtained according to the calculation formula of absorptivity *A* = 1 − |*S* | ^2^, as is shown in Fig. 5d. It can be seen that at a frequency of 94 GHz, the measured absorptivity is about 99.8%. Meanwhile, we tested the reproducibility of the experiment, and the details are described in supplementary S2. Therefore, the experimental and simulation results have good consistency, which demonstrated that the stacked meta-MEMS chip can achieve high absorption under ultra-thin conditions, laying the foundation for the next step of using visible light to achieve millimeter wave imaging.

### Imaging performance of the meta-MEMS chip

**Fig. 6:**
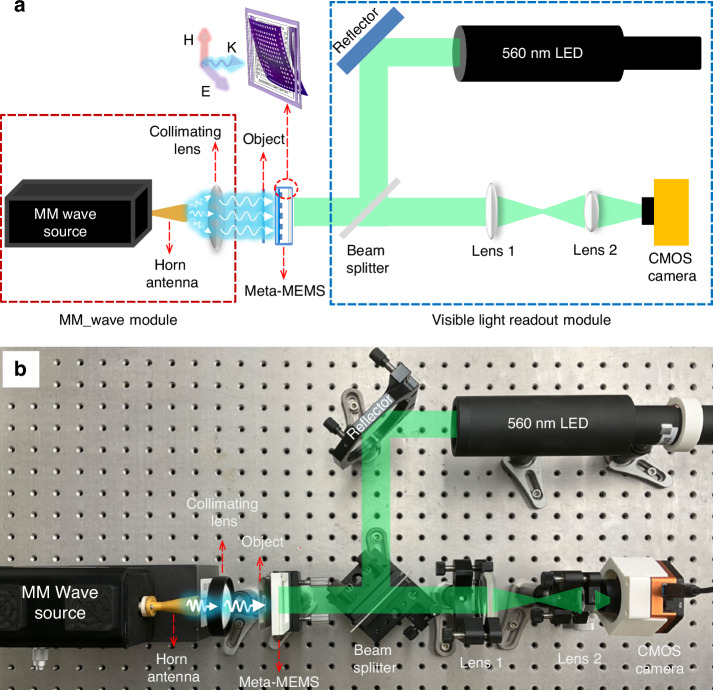
Imaging system. **a** Schematic diagram of the millimeter wave imaging system; **b** Photo of the millimeter wave imaging system

Figure 6a shows the schematic diagram of the millimeter wave imaging system. It mainly consists of two parts, which are the millimeter wave irradiation module and the visible light readout module. The millimeter wave irradiation module includes a millimeter wave source and a collimating lens. The visible light readout module includes a collimating LED source, a reflector, a beam splitter, an imaging lens group, and a CMOS camera. During the imaging process, the source emits millimeter waves, which pass through the collimating antenna and lens, and then irradiate onto the upper chip of the stacked meta-MEMS. The LED emits parallel light, which is reflected by the reflector and enters the beam splitter, then incident on the back of the lower chip, and illuminated to the micro-reflectors through the ITO glass substrate. When the chip absorbs millimeter wave radiation, its pixels will deflected. Under the action of micro-reflectors, the deflections of the pixels can be obtained. The light obtains the deflection information of the pixels and is focused by imaging lens 1 and lens 2. The two-dimensional distribution of the deflections of pixels represents the two-dimensional distribution of the detected millimeter waves. Then, the visible light CMOS camera obtains the distribution through the imaging lens group (lens 1 and lens 2), thereby achieving millimeter wave (MM_wave) imaging.

Figure 6b shows a photo of the millimeter wave imaging system. The MM_wave source is based on IMPATT diodes (the frequency is 94 GHz, and power is about 50 mW). The visible light source is a 560 nm LED, and the emitted light is collimated and expanded to become parallel light with a spot diameter of 50 mm. The CMOS sensor chip adopts SONY IMX174, whose effective sensitive size is 11.3 mm × 7.1 mm.

**Fig. 7:**
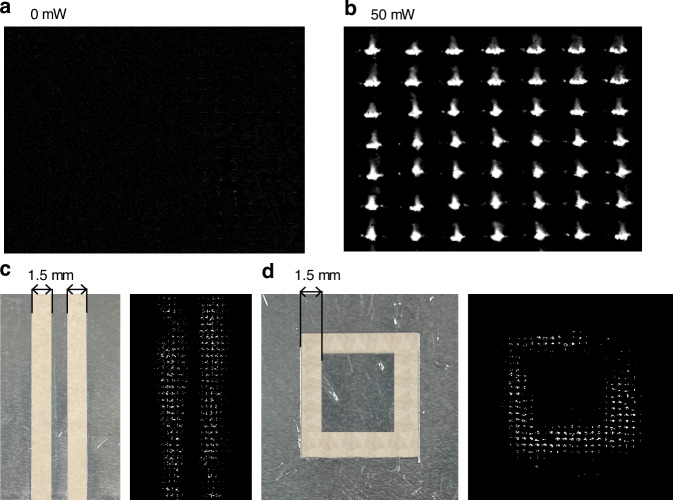
Imaging results. **a** The visual light readout imaging result without the millimeter wave irradiation; **b** The imaging result when the millimeter wave is irradiated on the 7 × 7 pixels area; **c** Imaging result of an aluminum plate with two hollow stripes; **d** Imaging result of an annular hollow aluminum plate

The experiments consist of two parts. The first part is to image the millimeter wave radiation without any objects; The second part is to image the actual objects under millimeter wave irradiation. Figures 7a and 7b show the first part experimental results. Among them, Fig. 7a is the visual light readout imaging result without the millimeter wave irradiation, and Fig. 7b is the imaging result when the millimeter wave is irradiated on a specified area (about 7 × 7 pixels) of the meta-MEMS chip. It can be seen that the pixels in this region have a significant radiation response. Meanwhile, the relationship between the MEMS deflection and millimeter wave power is simulated and analyzed, and the details are described in supplementary document S3.

Figure 7c, d show the second part of the experimental results. Among them, Fig. 7c is the imaging result of an aluminum plate with two hollow stripes, and Fig. 7d shows the imaging result of an annular hollow aluminum plate. It could be seen that the 94 GHz millimeter wave imaging system based on the meta-MEMS can obtain high-resolution images of 1.5 mm. Because the pixel size of the meta-MEMS chip is 984 × 716 µm, without considering the diffraction limitations of the lens, even an optimal resolution of 1 mm can be obtained theoretically.

**Fig. 8:**
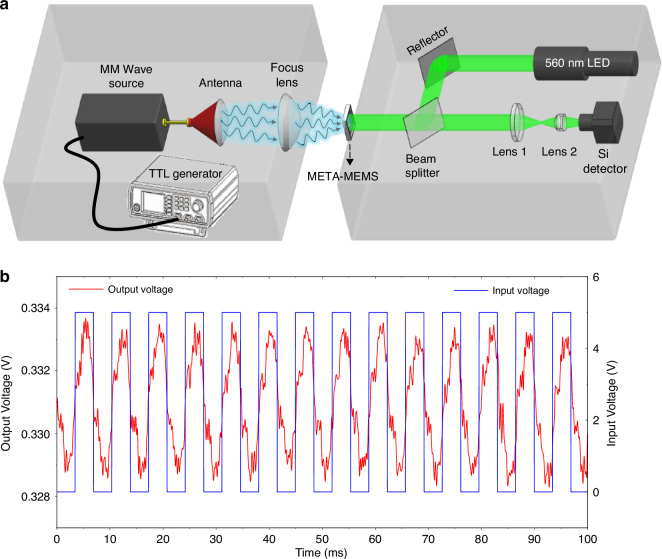
Response time test. **a** Schematic diagram of the response time testing system; **b** Curves of the input TTL pulse voltage and the optical readout signal voltage

Another important advantage of the stacked chip is that the meta-MEMS sensing and execution structure is very thin, at 1.2 microns, which is only 1/2500 of the wavelength. Moreover, the weight-reduction holes effectively reduce the weight of the chip, thus helping to achieve a higher response speed. Next, we tested the response time of the system. Figure 8a shows a schematic diagram of the response time measurement system. Similar to the optical readout experimental setup, it also consists of a millimeter wave source and a visible light readout module. The difference is that the millimeter wave source is modulated on and off by input of an external TTL pulse. In the experiment, we set the TTL modulation period to 144 Hz and then obtained the readout signal value through the optical readout module. It should be noted that in order to achieve faster signal measurement, a high-speed silicon detector is used instead of CMOS cameras. The test results are shown in Fig. 8b. The blue waveform in the Figure is the TTL pulse input by the signal generator, with a frequency of 144 Hz and an amplitude of 5 V. The red waveform represents the voltage value of the optical readout signal. It can be seen that the designed millimeter wave detector can respond to the switching modulation, and the response time of the system can be as fast as 7 ms. In addition, we have tested the NEP (Noise Equivalent Power) of the stacked chip, and the result is 2.5 × 10^−11^ W×Hz^−1/2^. The specific test method and test data are described in detail in Supplement S4.

## Discussion

The design, fabrication, and testing of a 94 GHz stacked meta-MEMS array chip have been reported. It combines the advantages of metamaterials and MEMS to construct an ultra-thin, high-performance meta-MEMS structure, and realize millimeter wave imaging using light. Different from the traditional receiver array-based electrical readout millimeter wave imaging devices^18,19^, and most metamaterial-enhanced millimeter wave imaging componets^20–22^. The meta-MEMS chip is based on the optical readout method, which can perfectly absorb electromagnetic waves, efficiently convert them into mechanical energy, and drive high-speed movement of optical micro-reflectors array, thereby achieving detection and imaging. It adopts the design of upper and lower chip separation and then stacking. The structure integrates micro-optics, micro-mechanics, and absorption units with a thickness less than λ/2500. The pixel size is less than λ/3. All the pixels do not need wires to connect, and the signal does not need wires to lead out. The increase in chip pixels does not increase the design complexity and preparation difficulty.

In order to verify the validity of the design and preparation methods of the meta-MEMS chip, we built a visible light readout experimental system and used light to image millimeter waves. The experimental results show that the absorptivity of the chip reaches 99.8% at 94 GHz, the number of pixels is 40 × 40, and the response time of the system is 7 ms (with a response speed of 144 Hz). They demonstrated the high performance of the meta-MEMS devices in the field of 94 GHz millimeter wave focal plane array imaging. In the future, with the optimization of structural design and improvement of preparation processes, sensitivity, and response speed may be further enhanced. Therefore, we believe that the design idea and imaging method adopted will be able to serve as a promising advanced technology platform for a wide range of millimeter wave detection and imaging applications.

## Materials and methods

### Simulation method

We designed and optimized the 94 GHz stacked meta-MEMS chip using electromagnetic simulation software CST 2023. In the simulation, the boundary conditions are set to unit cell in x and y directions and open (add space) in z direction based on the floquet mode. The tetrahedral meshing and frequency domain solver based on finite element method were used. In order to get the optimal structure size, we adopted the idea of two-step simulation (first rough simulation and then fine simulation). The specific optimization method is described in detail in the supplementary document S1. Next, the multi-physics simulation software COMSOL 6.1 was used to simulate and analyze the deformation of a single meta-MEMS pixel. We calculated the heat generated by millimeter wave incidence using the Electromagnetic wave Module, analyzed the thermal flow of the material through the Solid heat transfer Module, added constraint and free ends to the frame of the unit through the Solid mechanics module, and finally simulated the thermal-mechanical deformation of a single pixel through the multi-physics simulation.

### Fabrication method

**Fig. 9:**
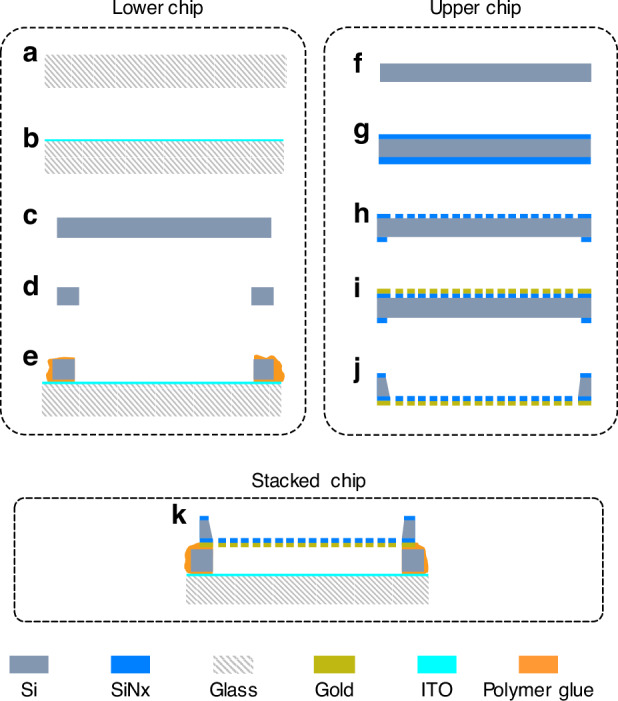
Process flow diagram. The process flow diagram: **a** cleaning glass surfaces; **b** Coating ITO film; **c** Silicon wafer thinning; **d** Cutting silicon wafer as support blocks; **e** Gluing the support block and ITO glass; **f** Double-sided polishing; **g** Double-sided SiNx deposition; **h** Photolithographed and etched; **i** Coating Au film and lifted off; **j** ICP etching and Wet etching the silicon substrate; **k** Stacking the upper and lower chips

A simple fabrication method is proposed for the processing of the meta-MEMS chips. Figure 9 shows the process flow diagram. The upper and lower chips are processed separately and then bonded into a stacked chip. The specific preparation process of the lower chip is as follows: (a) select quartz glass as a transparent substrate and clean its surfaces; (b) the glass is coated to form an ITO film with a square resistance of 6 ohms; (c) select a 2-inch silicon wafer with a thickness of 200 µm and thin it to the specified thickness by chemical mechanical polishing (CMP); (d) the silicon wafer is precisely cut into four silicon blocks by femtosecond laser; (e) the blocks is glued on the ITO glass substrate to form a silicon support frame to complete the fabrication of the lower chip.

The preparation process of the upper chip is as follows: (f) a silicon wafer with a thickness of 300 µm is selected for double-sided polishing. The double polishing technology is used to ensure the uniformity of back ICP silicon etching; (g) Perform double-sided SiNx deposition on the wafer with a thickness of 800 nm. The SiNx thin film was deposited using LPCVD technology, which has low stress and can serve as the underlying material for double-layer thin films, as well as a barrier material for silicon etching; (h) The top and bottom of the wafer are lithographed and etched to form a patterned silicon nitride structure; (i) The top of the wafer is photolithographed, coated with Au film, and lifted off to form a metal patterned structure, with an Au film thickness of 400 nm; (j) Dry etching (ICP) technology was used to etch the bottom of the wafer at a depth of 200 µm. Then, the silicon substrate is further etched using wet etching technology to penetrate the SiNx film, forming micro-reflectors array and bi-material cantilever structures as shown in Fig. 9j.

Finally, the upper and lower chips are glued together using polymer bonding technology to form a stacked meta-MEMS chip as shown in Fig. 9k. It should be mentioned that polymer bonding leads to an increase in thickness. We measured the thickness of the bonding layer and analyzed its influence on the electromagnetic characteristics of the chip. They are described in detail in the supplementary document S5.

## Supplementary information

Supplementary Information for Using light to image millimeter wave based on stacked meta-MEMS chip

## Data Availability

The authors declare that the data supporting the findings of this study are available with the paper and its Supplementary Information files. Meanwhile, the data that support the findings of this study are available from the corresponding author upon reasonable request.
