# Supplementary material for: Using light to image millimeter wave based on stacked meta-MEMS chip
Source: Light Sci Appl. 2025 Jan 22;14:59. doi: 10.1038/s41377-024-01733-6 (PMC11751477; doi:10.1038/s41377-024-01733-6)
Supplement: Supplementary file 1 — Supplementary Information for Using light to image millimeter wave based on stacked meta-MEMS chip [file 41377_2024_1733_MOESM1_ESM.docx]

**Supplementary Information for**

**Using light to image millimeter wave based on stacked meta-MEMS chip**

**Han Wang^2^, Zhigang Wang^2^, Cheng Gong^1,*^, Xinyu Li^1^, Tiansheng Cui^1^, Huiqi Jiang^1^, Minghui Deng^1^, Bo Yan^2^, and Weiwei Liu^1^**

*^1^Institute of Modern Optics, Nankai University, Tianjin Key Laboratory of Micro-scale Optical Information Science and Technology, Tianjin, 300350, China*

*^2^School of Electronic Science and Engineering, University of Electronic Science and Technology of China, Chengdu, 611731, China*

Han Wang 202111022724@std.uestc.edu.cn
Zhigang Wang zhigangwang@uestc.edu.cn
Xinyu Li [202411021619@std.uestc.edu.cn](mailto:202411021619@std.uestc.edu.cn)

Tiansheng Cui cuitiansheng@mail.nankai.edu.cn

Huiqi Jiang 2120230448@mail.nankai.edu.cn
Minghui Deng 2120230320@mail.nankai.edu.cn

Bo Yan yanbo@ee.uestc.edu.cn

Weiwei Liu [liuweiwei@nankai.edu.cn](mailto:liuweiwei@nankai.edu.cn)

**Corresponding author:**

Cheng Gong

Mail:[*gongcheng@nankai.edu.cn](mailto:*gongcheng@nankai.edu.cn)

[TEL: +86](TEL:+86) 13072069517

**Table of Contents**

**Section S1 : Structure and size optimization method of the meta-MEMS**

**Section S2 : The reproducibility of the experiments**

**Section S3 : The relationship between MEMS deflection and millimeter wave power**

**Section S4 : The NEP (Noise Equivalent Power) testing method and results**

**Section S5 :** **The thickness of polymer bonding layer and its influence on the chip**

**S1. Structure and size optimization method of the meta-MEMS**


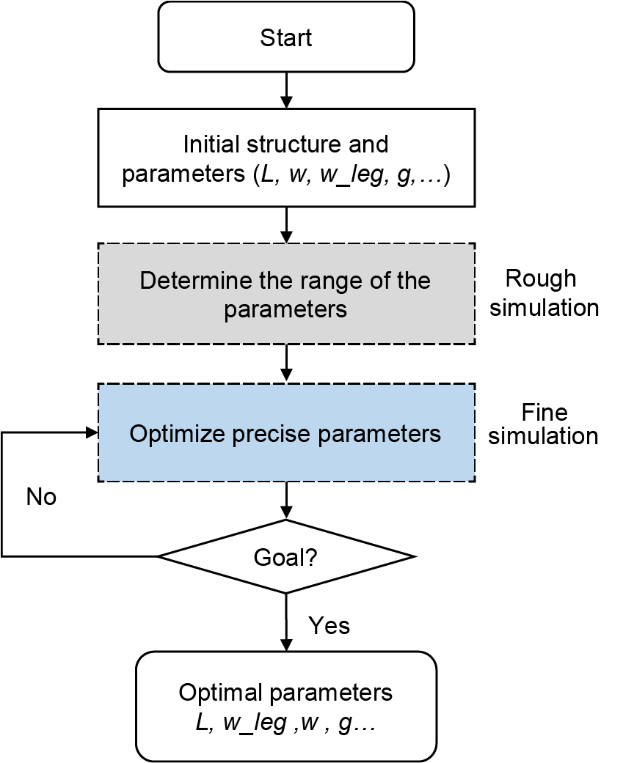


**Fig. S1.** The specific steps of optimization method.

The meta-MEMS could be regarded as an equivalent composite material. Due to the complex sub-wavelength structure, it is difficult to accurately calculate the absorption spectrum using analytical methods. Therefore, a numerical calculation method is used. Firstly, a three-dimensional physical model of the meta-MEMS pixel is established in electromagnetic simulation software, and the port, frequency, boundary conditions and simulation algorithm are set. Then the S-parameters are simulated to obtain the reflection coefficient (*S*_11_) and transmission coefficient (*S*_21_).

When optimizing the structure size, we adopted the idea of first rough simulation and then fine simulation. As shown in Figure S1, the specific optimization method is described as follow:

1) Select the initial structure and initial size parameters;

2) Rough simulation. Determine the parameters adjustment range preliminarily by parameter sweep;

3) Fine simulation. Optimize precise parameters according to the goal by multiple iterations;

4) Obtain the optimal size and parameters.

**S2. The reproducibility of the experiments**

We conducted repetitive experiments on the absorption rate and response time of the meta-MEMS chip. Among them, the absorption rate was measured for 5 times, and the results are shown in Figure S2-1. It can be seen that the absorption rate of each time is different, but it is above 99%, which proves that the repeatability meets the requirements.


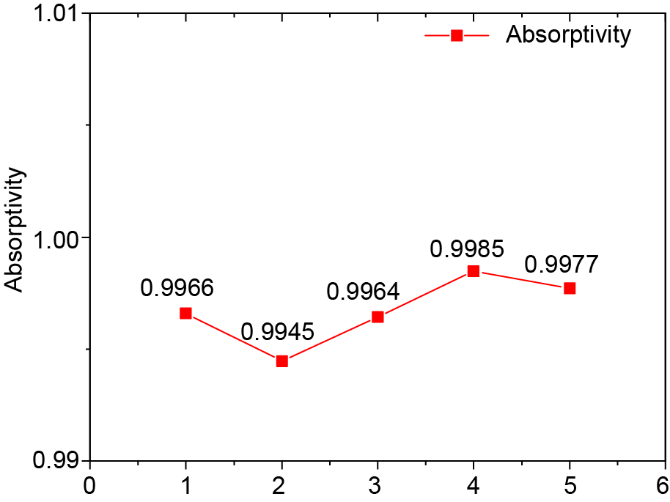


**Fig. S2-1.** Results of absorptivity repeatability experiment.

Next, we measured the response time of the chip three times. The response time curves obtained are shown in Figure S2-2 (a), and the filtered curves are shown in Figure S2-2 (b). The filtering method used is a low-pass filtering algorithm based on Fourier transform, which removes high-frequency noise and obtains relatively smooth response curves. It can be seen that the response time is about 7 ms each time, which proves its repeatability is also good.


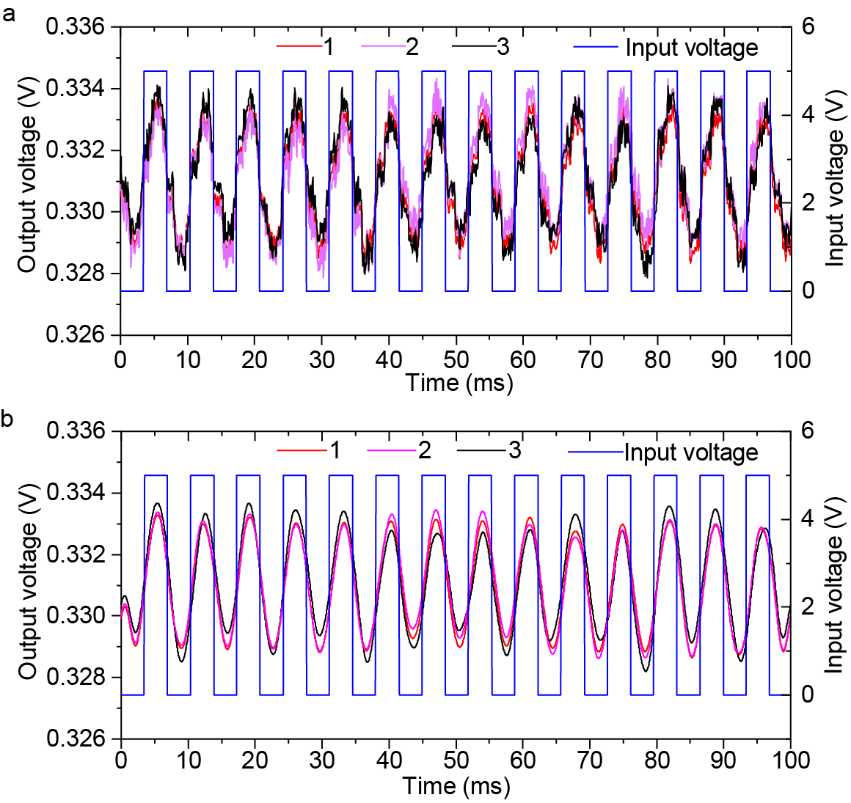


**Fig. S2-2.** (a) Results of response time repeatability experiment; (b) The Filtered results.

**S3. The relationship between MEMS deflection and millimeter wave power**

We analyzed the relationship between the MEMS cantilever deflection and the millimeter wave power from the following two aspects:

1) Is the deflection a function of millimeter wave power?

We used COMSOL multi-physics software to model and simulate the pixel of the meta-MEMS chip, and analyzed the relationship between equivalent incident power and deformation, as shown in Figure S3-1. It can be seen that the deformation is a function of the millimeter wave power, and the deformation gradually increases with the increase of the power. At present, the equivalent power incident on the chip is 0~250 µW, and the simulated deformation is 0~5.11 µm. It should be mentioned that power cannot increase infinitely. Continuing to increase to a certain extent may cause damage to MEMS pixels [1].

**References:**

[1] Gong C, Zhao YJ, Dong LQ, Hui M, Yu XM, Liu XH. The tolerable target temperature for bimaterial microcantilever array infrared imaging. *Opt Laser Technol* **45**, 545-550 (2013).


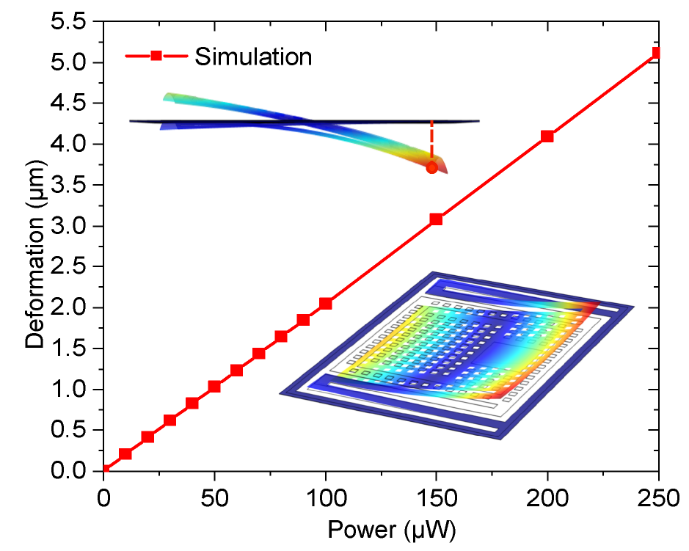


**Fig. S3-1.** Relationship between equivalent power and deformation.

2) Is it necessary to adjust the optical path as the power increases?

We analyzed the readout optical path using the geometric optics principle, as shown in Figure S3-2. When the meta-MEMS pixel deflects *θ*_0_, the reflected light will be deflected 2*θ*_0_, and the end point of the light incident to Lens 1 will be offset, from point A to point B, the distance traveled Δ*R* = *h* × tan(2*θ*_0_), where *h* is the distance from the meta-MEMS chip to the lens.

If Δ*R* is greater than the difference (Δ*k* ≈ 10.5 mm) between the radius of the lens and the pixel position at the edge of the chip, the light will not be received by the lens, so it is necessary to adjust the measurement optical path and use a lens with a larger radius. If Δ*R* is less than Δ*k*, the light can be received by the lens, so there is no need to adjust the measurement optical path.

We calculated the situation when light cannot be received: the deflection angle of the chip pixel is about 2 degrees and the deformation is about 12.5 µm. In this paper, the maximum power incident on the chip is 250 µW. By calculation, the deflection angle of the chip is about 0.8 degrees, and the deformation is only 5.11 µm. Because it is less than 12.5 µm, there is no need to adjust the measurement optical path.


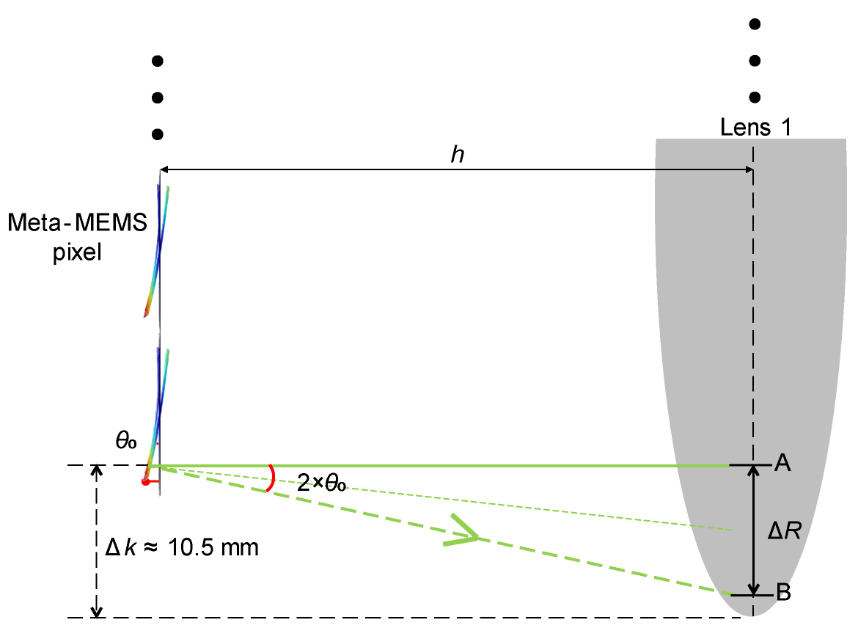


**Fig. S3-2.** Schematic of the readout optical path.

**S4. The NEP (Noise Equivalent Power) testing method and results**

We also benchmark the performance of the stacked meta-MEMS chip. We focused on the NEP (Noise Equivalent Power). The measuring system is shown in Figure S4-1, which mainly consists of a power adjustable millimeter wave module and a visible light readout module. The source emits millimeter waves, which pass through the collimating antenna, and then irradiate on the front of the stacked meta-MEMS chip. At the same time, The LED (560 nm) emits parallel light, which is reflected by a reflector and split by a beam splitter, and then incident on the back of the meta-MEMS chip. Based on the principle of optical leverage, the parallel light obtains the deflection information of the micro-mirrors on the meta-MEMS pixels, and is received by the silicon photodetector through the beam splitter and the focusing lens group (lens 1 and lens 2).

Then, the signal voltage value *V*_s_ of the photodetector is recorded by an oscilloscope, and the optical readout signal value *V*_o_ = *V*_s_-*V*_i_ can be obtained by subtracting the initial signal voltage value *V*_i_ (without radiation). When the power of the millimeter wave radiation to the chip gradually increased from low to high, we tested the voltage value *V*_s_, the initial voltage value *V*_i_, and the optical readout signal value *V*_o_.

It should be mentioned that, considering the loss of the millimeter-wave incident on the chip, when the power of the millimeter-wave source is increased from 5 mW to 100 mW, the actual equivalent power received by the meta-MEMS chip is 12.5 µW to 250 µW, as shown in Figure S4-2. The loss can be expressed as (1):

$$L=32.45+20\log(f)+20\log(d)$$

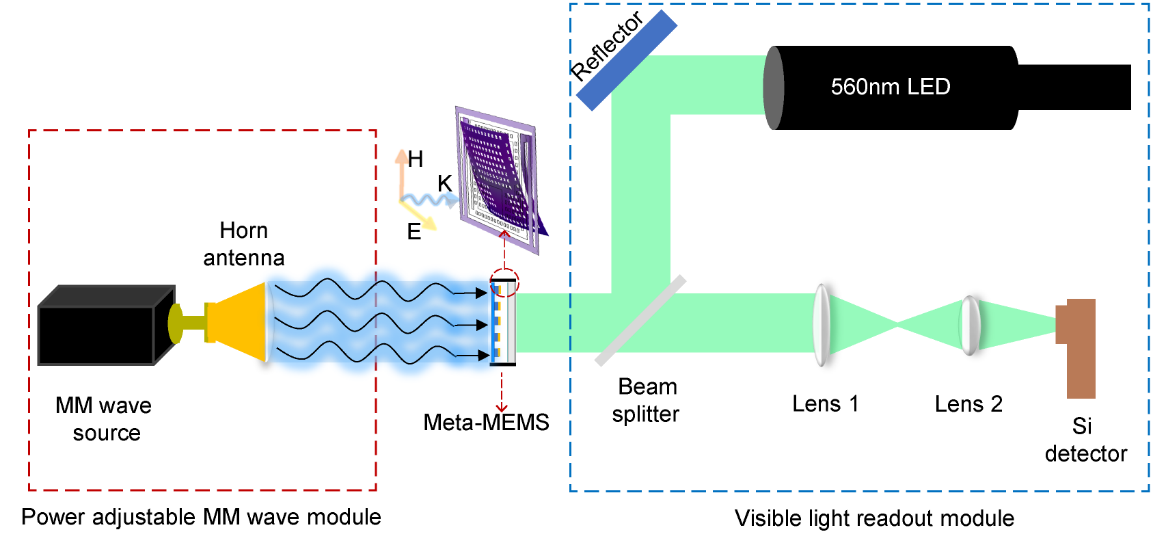


**Fig. S4-1.** Schematic diagram of the NEP measuring system.


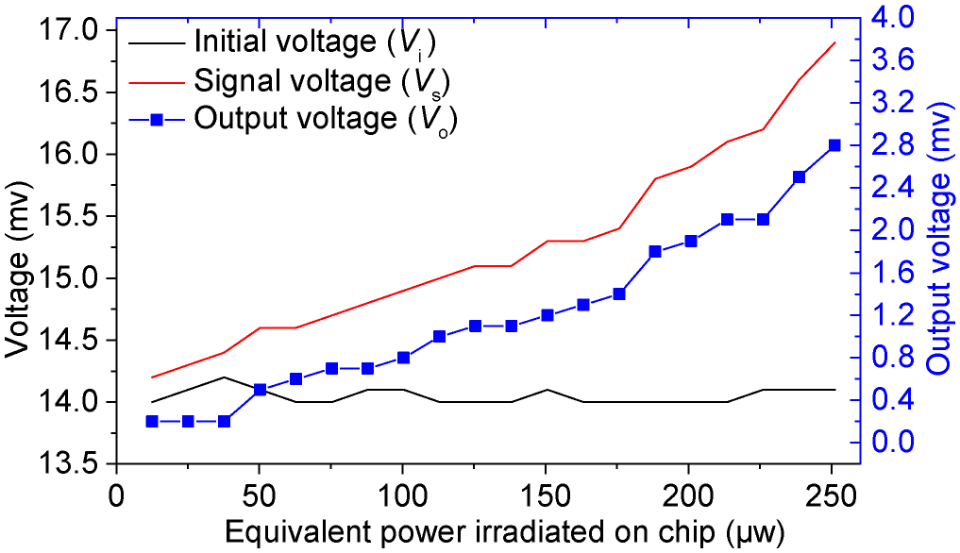


**Fig. S4-2.** Curves of the signal voltage value, initial voltage value and optical readout signal value changing with the equivalent power.

Where *f* is the frequency, *d* stands for the transmission distance. Through this formula, we can calculate that the loss is approximately 46 dB. The minimum power of the millimeter wave source is 5 mW (which is 7 dBm). Because the gain of the antenna is approximately 20 dB, the minimum power radiated to the metamaterial chip is -19 dBm (which is equivalent to 12.5 µW).

Next, the NEP could be calculated. As one of the most important metrics for the detectors, the NEP can be expressed as NEP = *N*/*R_v_*, where *N* is the noise of the system and *R_v_* stands for the responsivity. In the meta-MEMS chip, thermal noise is the main source of noise, so the *N* can be given by:

$$N=(4kTR_{d})^{1/2}$$

Here *k* is the Boltzmann constant, *T* is the temperature, and *R_d_* stands for the load resistance of the Si photodetector . Then, the *R*_v_ can be expressed as:

$$R_{v}=\frac{V_{o}}{P_{min}}$$

Where *P*_min_ is the minimum power received by the chip. *V_o_* represents the optical readout signal value. Accordingly, the NEP can be calculated as *N*/*R_v_* ≈ 2.5×10^-11^ W×Hz^-1/2^.

**References:**

[1] Pozar D M. Microwave Engineering, Fourth edition [J]. (2005).

**S5. The thickness of polymer bonding layer and its influence on the chip**

In the stacked meta-MEMS chip, the silicon support frame separates the upper chip and lower chip, creating the air gap layer. The frame consists of four silicon support blocks with customized thickness. Polymer bonding can lead to an increase in thickness, thereby affecting the thickness of the air layer, so it is necessary to determine the thickness of polymer bonding layer. We will analyze the thickness and its influence from the following aspects:


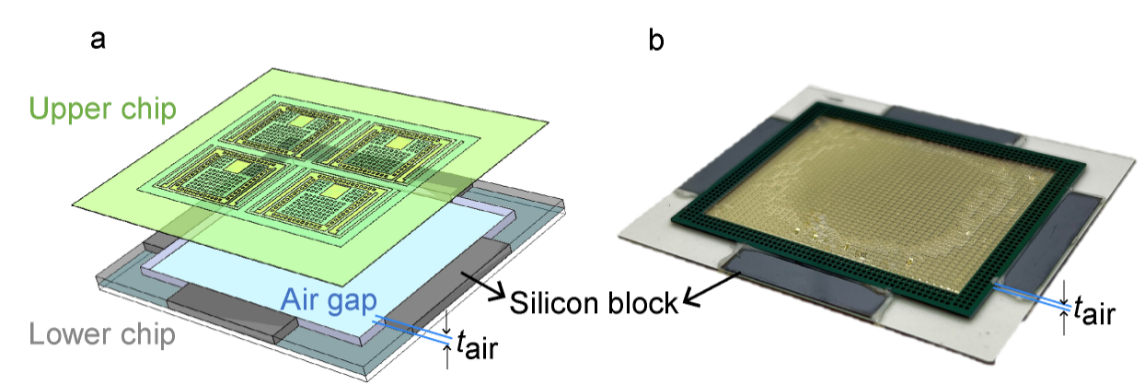


**Fig. S5-1.** (a) Schematic diagram of air gap layer and silicon support blocks; (b) photo of the stacked chip with silicon support blocks.

1) How to determine the thickness of the air layer?

The air thickness is determined by four customized thickness silicon support blocks, as shown in Figure S5-1 (a). The silicon blocks separate the upper chip and lower chip, creating the air gap layer. Figure S5-1 (b) is a photo of the stacked chip with silicon support blocks. It should be noted that the customized silicon block is thinned to 100 µm by chemical mechanical polishing (CMP), and then precisely cut by femtosecond laser. The size of each silicon block is 22 mm × 7 mm.

2) What is the thickness variation caused by the polymer bonding step?


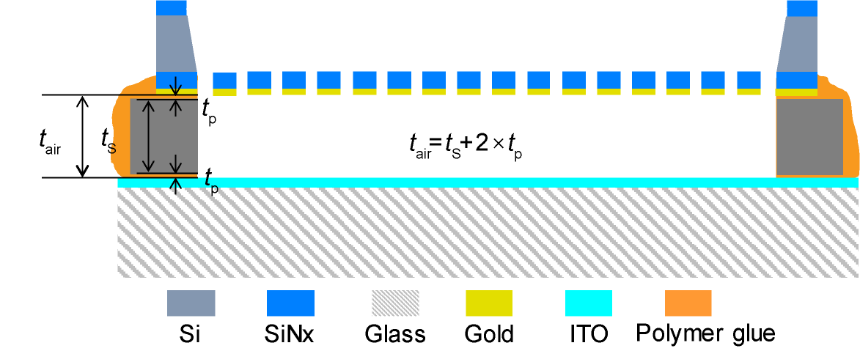


**Fig. S5-2.** Schematic diagram of the actual air layer thickness.

As shown in Figure S5-2, the actual air thickness (*t*_air_) should be the thickness of the silicon support block (*t*_s_) and the thickness of the polymer bonding layer (*t*_p_), which can be expressed as *t*_air_ = *t*_s_+2×*t*_p_.

To characterize the thickness variation of the polymer bonding step, we used the following method, as shown in Figure S5-3:

a) Measure the thickness (*t*_s_) of the silicon support block with a micrometer;

b) Measure the thickness (*t*_1_) of ITO coated glass substrate coated with a micrometer;

c) After bonding the silicon block and glass substrate, measure the overall thickness (*t*_2_) with a micrometer.

Finally, the thickness of the polymer can be obtained by the formula *t*_p_ = *t*_2_-*t*_1_-*t*_s_.


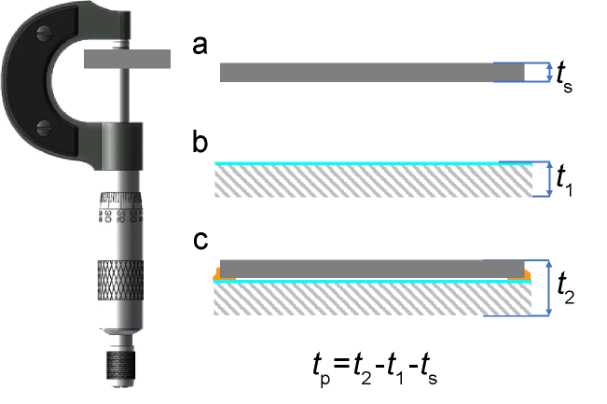


**Fig. S5-3.** Diagram of measuring polymer thickness with micrometer.

Through measurement, the thickness (*t*_p_) generated by single-layer polymer bonding is about 6 microns. After the upper and lower chips are bonded, the thickness change will be twice, that is, 2×*t*_p_ ≈ 12 microns. Therefore, the thickness variation caused by the polymer bonding step is about 12 µm.

3) Influence of the thickness variation on absorption of the stacked chip


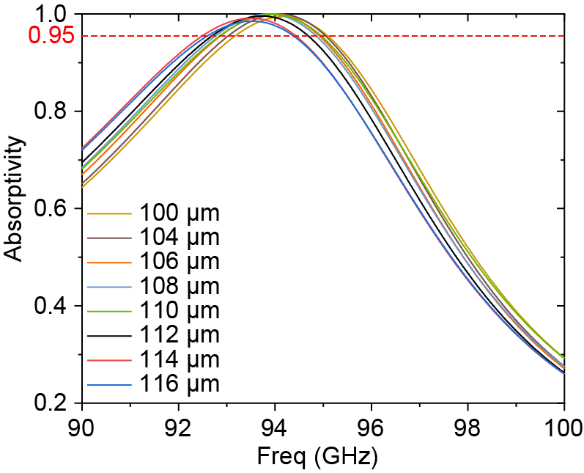


**Fig. S5-4.** Simulation results of absorption curve varying with air thickness.

The above measurements showed a significant (>10 µm) change in thickness after the polymer bonding. To analyze the effect of thickness variation on the absorption characteristics of the stacked chip, we conducted a series of simulations, as shown in Figure S5-4.

The thickness of the silicon support block is known to be 100 µm. Because the polymer bonding results in an increase (about 12 µm) in thickness, we simulated the absorption curves from 100 µm to 116 µm. It can be seen that within a certain range (≤16 µm), the change of air layer thickness (*t*_air_) does not have a significant impact on the absorption rate of the stacked chip, but only has a certain frequency shift.
